# Prior Immune Checkpoint Inhibitor Treatment Is a Risk Factor for Treatment‐Related Adverse Events in Unresectable Hepatocellular Carcinoma Treated With Durvalumab Plus Tremelimumab

**DOI:** 10.1002/jgh3.70163

**Published:** 2025-04-23

**Authors:** Naohiro Watanabe, Takashi Kobayashi, Michihiro Iwaki, Asako Nogami, Naohiro Wada, Ayako Shimizu, Tomoya Komori, Hirofumi Koike, Yukiko Sahashi, Atsushi Nakajima, Masato Yoneda

**Affiliations:** ^1^ Department of Pharmacy Yokohama City University Hospital Kanazawa Ward Japan; ^2^ Department of Gastroenterology and Hepatology Yokohama City University Hospital Kanazawa Ward Japan

**Keywords:** durvalumab, immune checkpoint inhibitors, treatment‐related adverse events, tremelimumab, unresectable hepatocellular carcinoma

## Abstract

**Aims:**

In March 2024, the American Society of Clinical Oncology recommended the combination of tremelimumab plus durvalumab as a treatment for advanced hepatocellular carcinoma (HCC). Although safety data for first‐line treatments are available, information on adverse events related to late‐line treatments is limited. This study aimed to identify risk factors for adverse events in patients who received this combination.

**Methods and Results:**

We conducted a retrospective cohort study from March 2023 to January 2025 at Yokohama City University Hospital, involving 24 patients aged 18 years or older with unresectable HCC. All 24 patients experienced at least one adverse event during treatment. Of these, the incidence of treatment‐related adverse events leading to treatment discontinuation after tremelimumab plus durvalumab therapy was 50.0% (12/24). In the discontinuation group, prior atezolizumab plus bevacizumab therapy (66.7% vs. 16.7%, *p* = 0.036) was more frequent than in the continuation group.

**Conclusion:**

In patients with unresectable HCC who received tremelimumab plus durvalumab, the risk of treatment‐related adverse events was associated with prior atezolizumab plus bevacizumab therapy. These factors may increase the likelihood of developing treatment‐related adverse events.

## 1 Introduction

Hepatocellular carcinoma (HCC) is a major global cause of morbidity and mortality, with a poor prognosis primarily due to high recurrence rates, chemotherapy resistance, and limited effective treatment options [1]. Specifically, patients diagnosed with unresectable HCC, a form of liver cancer that cannot be surgically removed, typically have limited treatment options. Systemic chemotherapy with sorafenib was initiated in 2007 [2, 3]. Since then, various multikinase inhibitors, such as lenvatinib [4], regorafenib, ramucirumab [5, 6], and cabozantinib, have been developed as systemic chemotherapy options for HCC. In 2020, atezolizumab (an anti‐PD‐L1 antibody) plus bevacizumab (an anti‐VEGF antibody) was introduced as immune checkpoint inhibitor (ICI) therapies [7]. In 2022, dual ICI therapy using durvalumab (an anti‐PD‐L1 antibody) plus tremelimumab (an anti‐CTLA‐4 antibody) was introduced. The HIMALAYA trial demonstrated that the combination of durvalumab plus tremelimumab reduced the risk of death by 22% compared to sorafenib and significantly increased the overall survival rate from 13.77 months to 16.43 months. In March 2024, the American Society of Clinical Oncology updated its guidelines for the systemic therapy of advanced HCC, recommending tremelimumab in combination with durvalumab as a first‐line treatment and as a second‐line treatment following atezolizumab plus bevacizumab [8]. The safety profile of tremelimumab combined with durvalumab in first‐line treatment aligns with the known safety profiles of each agent [9]. However, reports of adverse reactions related to subsequent lines of treatment following the combination of atezolizumab plus bevacizumab are limited. The inclusion criteria for the HIMARAYA trial targeted patients without prior systemic chemotherapy, and the adverse event profile following the combination of tremelimumab plus durvalumab in patients with a prior treatment history has not been confirmed. Therefore, we conducted a retrospective study to identify risk factors associated with treatment‐related adverse events in patients treated with the combination therapy of tremelimumab plus durvalumab.

## 2 Methods

### 2.1 Patient Information

We conducted a single‐center retrospective cohort study at Yokohama City University Hospital on patients aged 18 years or older, diagnosed with unresectable HCC between March 2023 and January 2025. These patients received combination therapy with tremelimumab plus durvalumab. The patients were classified according to the Barcelona Clinic Liver Cancer (BCLC) staging system, with a Child‐Pugh score of class A or B, a modified albumin‐bilirubin (mALBI) grade of 1a, 2a, or 2b, and a Performance Status (PS) score of 0 or 1 according to the Eastern Cooperative Oncology Group. No exclusion criteria were applied. This study was approved by the Institutional Review Board (Approval Number: F240400006) at Yokohama City University Hospital. Written informed consent was not obtained, as patient records and information were anonymized and analyzed.

### 2.2 Treatment

All patients were treated with the STRIDE (Single Tremelimumab Regular Interval Durvalumab) regimen, as used in the HIMARAYA trial. The STRIDE regimen consists of a single dose of 300 mg tremelimumab and 1500 mg durvalumab, followed by a single dose of 1500 mg durvalumab administered every 4 weeks. Discontinuation of treatment was determined by the treating physician based on the adverse event profile. Patients were monitored using routine laboratory tests, including hematology, biochemistry, and urinalysis, prior to the administration of tremelimumab plus durvalumab. Vital signs were assessed on the first day of each cycle, and computed tomography scans were performed every 2–3 months.

### 2.3 Evaluation of Clinical and Laboratory Values

Clinical data was collected during the first consultation, prior to the initiation of systemic therapy, and from medical records during scheduled follow‐up visits until January 2025. Vital signs, changes in clinical symptoms, potential adverse events related to antineoplastic medications, and hospitalizations were systematically documented. The attending physician assessed the Child‐Pugh score. The ALBI score was calculated using the following formula:

$$\begin{array}{c} \text{ALBI score}=\left(\log10\left(\text{total bilirubin}\;\left[\mathrm{mg}/\mathrm{dL}\right]\times 17.1\right)\times 0.66\right)\\ \;+\left(\text{albumin}\;\left[g/\mathrm{dL}\right]\times 10\times -0.085\right) \end{array}$$

mALBI grades were classified as follows: Grade 1 (good liver function) for ALBI scores ≤ − 2.6; Grade 2a (moderate hepatic impairment) for scores > − 2.6 and < −2.27; Grade 2b (moderate hepatic impairment) for scores ≥ − 2.27 and ≤ −1.39; and Grade 3 (reduced liver function) for scores > − 1.39. Treatment‐related adverse events were assessed according to the National Cancer Institute Common Terminology Criteria for Adverse Events, version 5.0. This classification divides severity into five grades: Grade 1 (mild), Grade 2 (moderate), Grade 3 (severe or medically significant, but not immediately life‐threatening), Grade 4 (life‐threatening), and Grade 5 (death).

### 2.4 Statistical Analysis

Patients were divided into two groups: the discontinuation group and the continuation group. The discontinuation group included patients who experienced Grade ≥ 3 treatment‐related adverse events following durvalumab plus tremelimumab therapy and were either admitted to the hospital or discontinued treatment at the physician's discretion. The continuation group included patients who discontinued durvalumab plus tremelimumab therapy due to disease progression by January 2025 and transitioned to other systemic chemotherapy, as well as those who discontinued treatment due to conversion surgery. The continuation group also included patients who experienced Grade ≥ 3 treatment‐related adverse events but were allowed to continue durvalumab plus tremelimumab therapy, with one patient being judged to have an adverse event as a result of an underlying disease.

Data were collected from 12 patients in the discontinuation group and 12 patients in the continuation group. Discrete variables were compared using Fisher's exact test, and continuous variables were compared using the Mann–Whitney *U* test. All *p* values were two‐sided, and a *p* value of < 0.05 was considered statistically significant. Data analysis was performed using IBM SPSS Statistics 27.

Sample size calculation was performed to ensure satisfactory power for Fisher's exact test. Assuming the event rate in the discontinuation group to be 60% and in the continuation group to be 10%, with the event rate in the continuation group based on the results of the HIMARAYA trial (which reported a treatment‐related adverse event rate of 8.2%), a significance level of 0.05, and a power of 80%, the required minimum number of patients per group was calculated. The calculated sample size for each group was approximately 18 patients.

## 3 Results

### 3.1 Response

**FIGURE 1:**
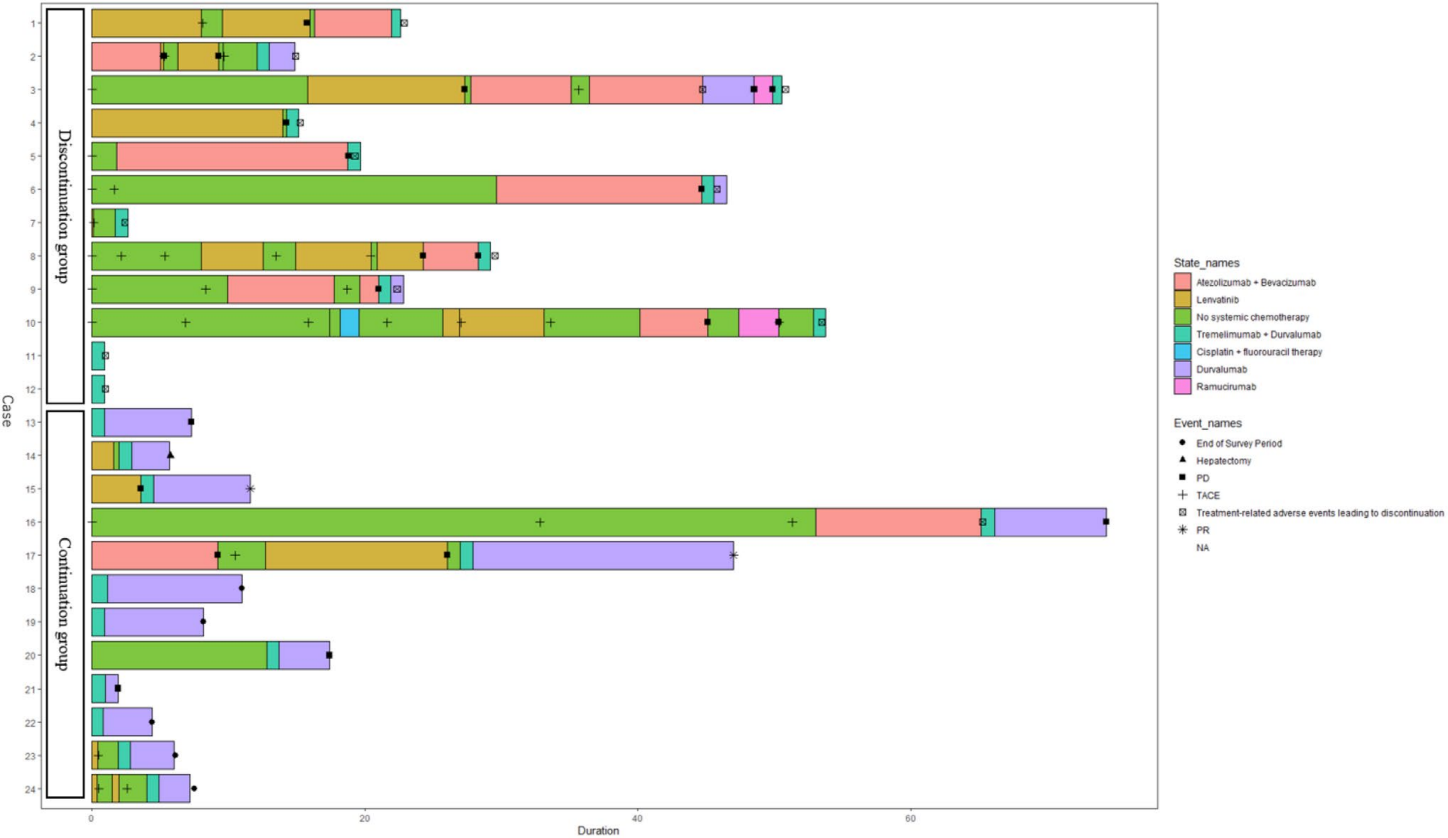
Swimmer plot for 24 eligible patients. Emerald color represents the treatment with tremelimumab plus durvalumab, purple represents cpresents the treatment with lenvatinib, salmon pink represents the combination therapy with あatezolizumab plus bevacizumab, green indicates no systemic chemotherapy, and pink represents the treatment with ramucirumab. Light blue represents the combination therapy of cisplatin plus 5‐Fluorouracil. One patient had esophageal cancer, so cisplatin plus 5‐Fluorouracil combination therapy was administered. ■ represents progression, + represents TACE (transarterial chemoembolization), ▲ represents liver resection, ☒ represents treatment‐related adverse events of Grade 3 or higher for durvalumab plus tremelimumab combination therapy, and ❋ represents Partial Response. The spiked circle indicates the end of the study period. Swimmer plots for all patients.

Twenty‐four patients were divided into two groups: the group that experienced treatment‐related adverse events leading to the discontinuation of durvalumab plus tremelimumab (*n* = 12) and the continuation group (*n* = 12) (Table 1, Figure 1). The incidence of treatment‐related adverse events leading to the discontinuation of durvalumab plus tremelimumab was 50% (12/24). One patient (8.3%) in the discontinuation group experienced Grade 5 adverse events related to aspartate aminotransferase (AST) and alanine aminotransferase (ALT) elevation. Of the two patients (16.6%) in the discontinuation group, one experienced Grade 4 colitis/diarrhea, and the other experienced Grade 4 treatment‐related adverse events related to AST/ALT elevation. Grade 3 treatment‐related adverse events occurred in nine patients (75%) in the discontinuation group. All patients (24/24) experienced at least one treatment‐related adverse event of any cause during at least one course of durvalumab plus tremelimumab therapy. The main symptoms included fatigue, diarrhea, blisters, adrenal insufficiency syndrome, elevated bilirubin, pneumonia, and AST/ALT elevation. The most common Grade 1 or 2 treatment‐related adverse events due to any cause in the discontinuation group included fatigue, anorexia, elevated creatine kinase, skin conditions (erythema multiforme, pruritus, hand‐foot syndrome, rash‐like skin), dysgeusia, and hyponatremia. In the continuation group, Grade 1 or 2 treatment‐related adverse events due to any cause included fatigue, pruritus, adrenal insufficiency syndrome, elevated AST/ALT, elevated bilirubin, constipation, albumin reduction, hyponatremia, and increased blood pressure.

**TABLE 1:** Any adverse events after durvalumab plus tremelimumab combination in the continuation and discontinuation groups.

	Discontinuation group (*n* = 12)					Continuation group (*n* = 12)				
	Grade 1	Grade 2	Grade 3	Grade 4	Grade 5	Grade 1	Grade 2	Grade 3	Grade 4	Grade 5
Patients with treatment‐related adverse events	9 (75%)	7 (58.3%)	9 (75%)	2 (16.7%)	1 8.3%)	11 (91.7%)	8 (66.6%)	1 (8.3%)	0	0
Fatigue	4 (33.3%)	1 (8.3%)	1 (8.3%)	0	0	5 (41.7%)	0	0	0	0
Diarrhea	0	0	2 (16.7%)	1 (8.3%)	0	0	0	0	0	0
Anorexia	1 (8.3%)	3 (25%)	0	0	0	0	0	0	0	0
Nausea	0	1 (8.3%)	0	0	0	0	0	0	0	0
Joint pain/muscle pain	2 (16.7%)	1 (8.3%)	0	0	0	0	0	0	0	0
Hypoadrenal gland function	0	0	1 (8.3%)	0	0	2 (16.7%)	0	0	0	0
Hypothyroidism	0	1 (8.3%)	0	0	0	1 (8.3%)	2 (16.7%)	0	0	0
Dry mouth	1	1	0	0	0	0	0	0	0	0
Mouth mucositis	0	1	0	0	0	0	0	0	0	0
Increased creatinine	1		0	0	0	1		0	0	0
Increased alanine transferase	0	2 (16.7%)	1 (8.3%)	21 (8.3%)	1 (8.3%)	1	2	0	0	0
Increased aspartate aminotransferase	0	1 (8.3%)	1 (8.3%)	21 (8.3%)	1 (8.3%)	2	2	0 (8.3%)	0	0
Increased alkaline phosphatase	0	0	0	0	0	1 (8.3%)	0	1 (8.3%)	0	0
Increased **γ**‐glutamyl transferase	0	0	0	0	0	0	0	1 (8.3%)	0	0
Taste disorders	2	1	0	0	0	0	0	0	0	0
Rash	0	0	0	0	0	0	0	0	0	0
Hyponatremia	3 (25%)	3 (25%)	0	0	0	5 (41.7%)	0	0	0	0
Hyperkalemia	1 (8.3%)	0	0	0	0	0	0	0	0	0
Hypomagnesemia	1 (8.3%)	0	0	0	0	0	0	0	0	0
Colitis	0	0	1 (8.3%)	1 (8.3%)	0	0	0	0	0	0
Elevated creatine kinase	1 (8.3%)	0	0	0	0	2 (16.7%)	1 (8.3%)	0	0	0
Constipation	1 (8.3%)	0	0	0	0	0	2 (16.7%)	0	0	0
Hand‐foot syndrome	2 (16.7%)	1 (8.3%)	0	0	0	0	0	0	0	0
Stomatitis	0	1 (8.3%)	0	0	0	0	0	0	0	0
Acne‐like skin rashes	1 (8.3%)	2	0	0	0	0	0	0	0	0
Erythema multiforme	0	1 (8.3%)	0	0	0	1 (8.3%)	0	0	0	0
Purpura	1 (8.3%)	0	0	0	0	0	0	0	0	0
Pruritus	2	1 (8.3%)	0	0	0	2	1 (8.3%)	0	0	0
Blosser	0	0	1 (8.3%)	0	0	0	0	0	0	0
Eczema	0	0	0	0	0	2	1	0	0	0
Changing the color of nails	1 (8.3%)	0	0	0	0	0	0	0	0	0
Elevated bilirubin	0	0	1 (8.3%)	0	0	0	2 (16.7%)	0	0	0
Pneumonia	0	0	1 (8.3%)	0	0	0	0	0	0	0
Lymphopenia	1 (8.3%)	0	0	0	0	1 (8.3%)	0	0	0	0
Increase in cerebral natriuretic peptide	0	0	0	0	0	1 (8.3%)	0	0	0	0
Increased blood sugar levels	1 (8.3%)	0	0	0	0	2 (16.7%)	0	0	0	0
Albumin lowering	0	1	0	0	0	0	3 (25%)	0	0	0
Hemoglobin lowering	0	0	0	0	0	0	1 (8.3%)	0	0	0
Eosinophilia	0	0	0	0	0	1 (8.3%)		0	0	0
Thrombocytopenia	0	1	0	0	0	1 (8.3%)		0	0	0
Increased pleural effusion	0	0	0	0	0	0	2 (16.7%)	0	0	0
Increased ascites	0	0	0	0	0	0	1 (8.3%)	0	0	0
Lower leg edema	0	0	0	0	0	2 (16.7%)	1 (8.3%)	0	0	0
Cough	0	0	0	0	0	1 (8.3%)	0	0	0	0
Fever	0	0	0	0	0	1 (8.3%)	1 (8.3%)	0	0	0
Protein in the urine	0	0	0	0	0	1 (8.3%)	0	0	0	0
Increased amylase	0	0	0	0	0	1 (8.3%)	0	0	0	0
Increased blood pressure	0	0	0	0	0	2 (16.7%)	0	0	0	0
Rotational vertigo	0	1 (8.3%)	0	0	0	1 (8.3%)	0	0	0	0
Hepatic hemorrhage	0	0	0	0	0	0	0	1 (8.3%)	0	0
Activated Partial Thromboplastin Time extended	0	0	0	0	0	1 (8.3%)	0	0	0	0

*Note:* The data is *n* (%). Adverse events were assessed according to the National Cancer Institute Common Terminology Criteria for Adverse Events version 5.0. This classification classifies the severity into five grades: mild Grade 1, moderate Grade 2, Grade 3 severe or medically significant but not immediately life‐threatening, life‐threatening Grade 4, and death Grade 5.

### 3.2 Survey Factor Results

The discontinuation/continuation group consisted of the following distributions: sex (Male: 9/11), PS (0, 1, 2) (9, 3, 0/7, 4, 1), Child‐Pugh classification (A5, A6, B7) (9, 3, 0/5, 6, 1), and mALBI grade (1, 2a, 2b) (5, 3, 4/5, 0, 7). Background liver disease included hepatitis B virus (HBV), hepatitis C virus (HCV), and non‐viral causes (6, 0, 6/3, 4, 5), large vessel infiltration (presence or absence) (1, 11/2, 10), and extrahepatic metastases (presence) (6, 6/5, 7). AFP > 400 (above/non‐it) was noted (6, 6/4, 8) (Table 2).

**TABLE 2:** Patient demographics and baseline characteristics.

	Discontinuation group (*n* = 12)		Continuation group (*n* = 12)		*p*
Age	69	(58, 78)	66.5	(54, 83)	0.932
Sex	9	(75%)	11	(91.7%)	0.59
Weight	56.4	(47.5, 105.2)	66.6	(48.9, 79.0)	0.41
ECOG performance status					
0	9	(75%)	7	(58.3%)	0.667
1	3	(25%)	4	(33.3%)	1
2	0		1	(8.3%)	1
Child‐Pugh classification					
A/5	9	(75%)	5	(41.7%)	0.214
A/6	3	(25%)	6	(50%)	0.4
B/7	0		1	(8.3%)	1
mALBI Grade					
Grade 1	5	(41.7%)	5	(41.7%)	1
Grade 2a	3	(25%)	0	(0%)	0.217
Grade 2b	4	(33.3%)	7	(58.3%)	0.414
HBV	6	(50%)	3	(25%)	0.4
HCV	0	(0%)	4	(33.3%)	0.093
Non‐Virus	6	(50%)	5	(41.7%)	0.682
Large vascular infiltration	1	(8.3%)	2	(16.7%)	1
Extrahepatic metastases	6	(50%)	5	(41.7%)	1
AFP ≧ 400	6	(50%)	4	(33.3%)	0.682
DCP	631.5	(21, 389398)	240.5	(21, 551070)	0.671

*Note:* The data is *n* (%) or Median (minimum, maximum).

Abbreviations: DCP, Des‐gamma‐carboxy‐prothrombin; ECOG, eastern cooperative oncology group.

Regarding prior systemic chemotherapy, treatment history was reported with or without (10, 2/6, 6). The history of atezolizumab plus bevacizumab therapy (with or without) was as follows: atezolizumab plus bevacizumab within 1 year (8, 4/2, 10). The history of bevacizumab therapy was (8, 4/1, 11), the history of lenvatinib therapy was (7, 5/5, 7), and 3 years of lenvatinib treatment was (7, 5/4, 8). The presence or absence of lenvatinib within 1 year was (4, 8/4, 8), and transcatheter arterial chemoembolization (TACE) (9, 3/4, 8). The presence or absence of TACE therapy within 3 years was (8, 4/4, 8), and the presence or absence of TACE therapy within 1 year was (5, 7/2, 10).

In the univariate analysis, the following were more frequently seen in the discontinuation group: atezolizumab plus bevacizumab (66.7% vs. 16.7%, *p* = 0.036), atezolizumab plus bevacizumab therapy within 3 years (66.7% vs. 8.3%, *p* = 0.009), and prior history of atezolizumab plus bevacizumab therapy within 1 year (66.7% vs. 8.3%, *p* = 0.009) (Table 3).

**TABLE 3:** Patient's previous treatment history at the time of introduction of durvalumab plus tremelimumab combination therapy.

	Discontinuation group (*n* = 12)		Continuation group (*n* = 12)		*p*
History of prior systemic chemotherapy	10	(83.3%)	6	(50%)	0.193
Previously treated with atezolizumab plus bevacizumab	8	(66.7%)	2	(16.7%)	0.036*
History of atezolizumab plus bevacizumab within 3 years	8	(66.7%)	1	(8.3%)	0.009*
History of atezolizumab plus bevacizumab within 1 year	8	(66.7%)	1	(8.3%)	0.009*
Previously received lenvatinib	7	(58.3%)	5	(41.7%)	0.684
Patients with a history of lenvatinib within 3 years	7	(58.3%)	4	(33.3%)	0.414
Prior history of lenvatinib within 1 year	4	(33.3%)	4	(33.3%)	1
There is a history of TACE	9	(75%)	4	(33.3%)	0.100
TACE implementation history is less than 3 years old	8	(66.7%)	4	(33.3%)	0.220
TACE implementation history is less than 1 year old	5	(41.7%)	2	(16.7%)	0.371

*Note:* The data is *n* (%).

* *p* < 0.05.

### 3.3 Treatment of Adverse Events

All patients who experienced treatment‐related adverse events were diagnosed with Grade 3 or higher, and 75% (9/12) required hospitalization. Patients with Grade 3 or higher adverse events developed these events within 3 doses of the STRIDE regimen (Table 4).

**TABLE 4:** Details of the 24 study participants.

	Case	Treatment‐related adverse event name	Number of cycles of Dur + Tre^a^	age	sex	Child‐pugh Classification	HBV	HBs antigens	HBC antibodies	HBV‐DNA (IU)	HCV antibodies	Large vascular infiltration	Extrahepatic metastases	AFP (ng/mL)	DCP (mAU/mL)
Discontinuation group	1	Blister certificate	1	56	M	A (6)	‐	‐	‐	N	‐	No	No	3	234
	2	Hepatic impairment	3	74	F	A (6)	‐	‐	‐	NA	‐	No	Yes	707	11 721
	3	Interstitial pneumonia	1	67	M	A (5)	+	+	+	Undetected	‐	No	Yes	4293	389 398
	4	Rash, liver disorder, hypopituitarism, decreased platelets, tenonitis	1	71	M	A (5)	+	+	+	4.37	‐	No	No	571	4891
	5	Enteritis	1	78	M	A (5)	‐	‐	‐	NA	‐	No	No	8	21
	6	Fatigue	2	64	M	A (5)	+	‐	+	NA	‐	No	No	3	554
	7	Enteritis	1	65	M	A (5)	+	+	+	< 1.00	‐	No	Yes	356	37 063
	8	Impaired liver function	1	58	F	A (5)	+	+	‐	3.01	‐	No	No	2161	1698
	9	Malaise, pituitary insufficiency	2	73	M	A (5)	+	+	+	1.75	‐	No	Yes	1115	709
	10	Enteritis	1	75	M	A (5)	‐	‐	+	Undetected	‐	No	Yes	10 535	282
	11	Hepatic impairment	1	78	F	A (5)	‐	‐	‐	NA	‐	Yes	Yes	15	49
	12	Hepatic impairment	1	59	M	A (6)	‐	‐	‐	NA	‐	No	No	12	390
Continuation group	13	No	6	68	M	A (5)	‐	‐	‐	NA	‐	No	No	3	64
	14	No	4	75	F	A (6)	‐	‐	+	Undetected	+	Yes	No	238 393	41 614
	15	No	7	55	M	A (6)	‐	‐	‐	Undetected	+	No	Yes	12 352	551 070
	16	No	9	78	M	A (5)	‐	‐	+	Undetected	+	No	No	18	293
	17	No	19	54	M	A (6)	‐	‐	‐	NA	‐	No	Yes	549	739
	18	No	12	62	M	B (7)	‐	‐	‐	Undetected	+	No	Yes	1	47
	19	No	10	63	M	A (5)	+	+	+	Undetected	‐	No	Yes	20 807	188
	20	No	4	83	M	A (6)	‐	‐	+	Undetected	‐	No	No	5	50
	21	No	2	82	M	A (6)	+	+	+	6.04	‐	No	No	128	125 247
	22	No	5	65	M	A (5)	+	+	‐	Undetected	‐	No	Yes	4	21
	23	No	4	59	M	A (6)	‐	‐	‐	NA	‐	No	No	12	85
	24	No	4	72	M	A (5)	‐	‐	‐	NA	‐	Yes	No	2	11 847

^a^ Dur + Tre:Durvalumab plus tremelimumab; DCP, Des‐gamma‐carboxy‐prothrombin.

All patients who required hospitalization were treated with steroids. One patient died as a result of treatment‐related adverse events. Due to treatment‐related adverse events, none of the patients resumed durvalumab plus tremelimumab therapy during the post‐study discontinuation period. In the continuation group, five patients discontinued treatment due to disease progression, two patients had a partial response, one patient underwent conversion surgery, and four patients exceeded the study period.

## 4 Discussion

In this study, treatment‐related adverse events of Grade 3 or higher occurred, and at the discretion of the physician, 50% of patients discontinued durvalumab plus tremelimumab therapy. The HIMARAYA trial, a phase III, randomized, open‐label, global, multicenter study on unresectable HCC, reported that the incidence of treatment‐related adverse events with durvalumab plus tremelimumab was 8.2%. However, in our study, the incidence of treatment‐related adverse events was significantly higher in the patient population. Our study identified a history of atezolizumab and bevacizumab therapies as risk factors for treatment‐related adverse events in patients receiving the durvalumab plus tremelimumab combination. Based on these results, careful attention to the patient's treatment history is essential when initiating combination therapy with tremelimumab plus durvalumab. Furthermore, most treatment‐related adverse events leading to discontinuation occurred within 3 months of initiating the STRIDE regimen [10]. In our study as well, the timing of treatment‐related adverse events leading to discontinuation was consistent, with all cases occurring within 3 months.

Patients included in the HIMARAYA trial had no prior treatment history. In our study, 66.7% of patients had received prior systemic chemotherapy, and 83.3% of the patients who discontinued durvalumab plus tremelimumab therapy due to treatment‐related adverse events had received prior systemic chemotherapy. Our study suggests that a history of atezolizumab plus bevacizumab therapy may be a risk factor for treatment‐related adverse events leading to the discontinuation of durvalumab plus tremelimumab therapy. Lenvatinib, a tyrosine kinase inhibitor, was not identified as a risk factor. These findings indicate that introducing the durvalumab plus tremelimumab combination therapy in patients with a history of ICIs may increase the incidence of Grade 3 or higher treatment‐related adverse events, which could lead to therapy discontinuation. ICIs require long‐term monitoring of side effects [11, 12], and the high incidence of treatment‐related adverse events suggests that careful pre‐treatment evaluation should be conducted before using durvalumab plus tremelimumab as a second‐line treatment. The American Association for the Study of Liver Disease (AASLD) guidelines recommend lenvatinib, sorafenib, regorafenib, ramucirumab, and cabozantinib as second‐line treatments when atezolizumab plus bevacizumab therapy is selected as the first‐line treatment. Durvalumab plus tremelimumab combination therapy is not recommended as a second‐line treatment following atezolizumab plus bevacizumab. In the development of treatment strategies, the effectiveness of treatment and the management of side effects should be carefully considered [13].

In our study, patients with HCV infection were not included in the discontinuation group and were only included in the continuation group. Direct‐acting antiviral (DAA) treatment for HCV causes significant changes in immunological factors, including the suppression of endogenous interferon (IFN) [14]. DAA therapy inhibits the activation of natural killer cells [15], maintains immunosuppression by regulatory T cells, and increases vascular endothelial growth factor and angiopoietin‐2, both of which are involved in angiogenesis. ICIs for hepatitis‐derived solid tumors have been shown to have a low incidence of Grade 3 or higher immune‐related adverse events [16]. In our study, no Grade 3 or higher treatment‐related adverse events were observed in patients with HCV‐derived HCC. Furthermore, in our study, a case of conversion was observed in HCV‐derived HCC patients who received DAA treatment followed by durvalumab plus tremelimumab therapy. Therefore, it is unlikely that immunosuppression after DAA treatment reduced the responsiveness to ICI treatment. Future studies are expected to further investigate the treatment responses and adverse event profiles of durvalumab plus tremelimumab in HCV‐derived HCC patients after DAA treatment.

In our study, the history of TACE tended to be higher in the discontinuation group. TACE is the standard of care for intermediate HCC that converts tumors into immune tumors by increasing tumor‐infiltrating lymphocytes and decreasing regulatory T cells and PD‐L1‐positive T cells due to inflammation of the tumor microenvironment after TAE/TACE [17, 18]. The combination of TAE/TACE and immunotherapy is compatible and can be expected to have a synergistic effect. The safety of TACE with ICIs and tyrosine kinase inhibitors provides significant clinical benefits for tumor response and survival without increasing the risk of serious adverse events [19]. Durvalumab plus tremelimumab therapy in immune hot tumor conditions can cause an excessive immune response, and evidence is expected to accumulate in the future.

There were several limitations in this study. The sample size calculation indicated that 18 participants per group and a total of 36 participants were needed. However, due to the single‐center retrospective design and the limited number of first‐line treatment cases, the required sample size could not be achieved. Additionally, because it was a retrospective study, it was not possible to definitively determine whether the adverse events that occurred during the study were immune‐related adverse events (irAEs). The study duration was insufficient, which led to inadequate evaluation of long‐term treatment effects and side effects. Furthermore, due to the small sample size, only univariate analysis was performed, and a more detailed examination using multivariate analysis could not be conducted. Additionally, the lack of survival data was another limitation. These constraints need to be addressed in future research to improve and obtain more reliable results.

The strength of this study is that it suggests that the administration of durvalumab plus tremelimumab combination therapy may increase the incidence of immune‐related adverse events in patients with a history of atezolizumab plus bevacizumab therapy. In the present study, one patient died as a result of treatment‐related adverse events following durvalumab plus tremelimumab therapy; however, many patients received alternative treatments after such events.

Administration of ICIs is more effective in HBV‐derived HCC than in non‐viral and HCV‐derived cases [20]. Patients who experience immune‐related adverse events are more likely to achieve an objective tumor response than those who do not experience immune‐related adverse events [21]. The management of adverse events associated with durvalumab plus tremelimab therapy is expected to be effective, and evidence is accumulating regarding the future treatment prognosis.

In patients with malignant melanoma, the prognosis is favorable if ICIs cause immune‐related adverse events. However, the adverse events of combination therapy with durvalumab plus tremelimumab should be adequately controlled. Further studies on the prognosis of treatment are needed.

## 5 Conclusion

In patients with unresectable HCC who received durvalumab plus tremelimumab combination therapy, the expression of immune‐related adverse events is associated with prior treatment with ICIs. Patients with this background may be at increased risk of developing treatment‐related adverse events with durvalumab plus tremelimumab therapy.

## Conflicts of Interest

The authors declare no conflicts of interest.
